# Identification of Free Nitric Oxide Radicals in Rat Bone Marrow: Implications for Progenitor Cell Mobilization in Hypertension

**DOI:** 10.1371/journal.pone.0057761

**Published:** 2013-03-12

**Authors:** Marina A. Aleksinskaya, Ernst E. H. van Faassen, Jelly Nelissen, Ben J. A. Janssen, Jo G. R. De Mey, Roeland Hanemaaijer, Ton Rabelink, Anton Jan van Zonneveld

**Affiliations:** 1 Department of Nephrology and the Einthoven Laboratory for Experimental Vascular Medicine, Leiden University Medical Center, Leiden, The Netherlands; 2 Department of Pharmacology, Cardiovascular Research Institute Maastricht, Maastricht University, Maastricht, The Netherlands; 3 TNO, Metabolic Health Research, Leiden, The Netherlands; Pennington Biomedical Research Center, United States of America

## Abstract

Nitric oxide (NO) has been implicated in matrix metallopeptidase 9 (MMP9)-dependent mobilization of hematopoietic stem and progenitor cells from bone marrow (BM). However, direct measurement of NO in the BM remained elusive due to its low *in situ* concentration and short lifetime. Using NO spin trapping and electron paramagnetic resonance (EPR) spectroscopy we give the first experimental confirmation of free NO radicals in rodent BM. NO production was quantified and attributed to enzymatic activity of NO synthases (NOS). Although endothelial NOS (eNOS) accounts for most (66%) of basal NO, we identified a significant contribution (23%) from inducible NOS (iNOS). Basal NO levels closely correlate with MMP9 bioavailability in BM of both hypertensive and control rats. Our observations support the hypothesis that inadequate mobilization of BM-derived stem and progenitor cells in hypertension results from impaired NOS/NO/MMP9 signalling in BM, a condition that may be corrected with pharmacological intervention.

## Introduction

Proper endothelial function is crucial for vascular homeostasis. Onset and progression of cardiovascular disease is characterized by an imbalance in the endothelial cell (EC) damage and repair [1]. Lost EC are replaced either by mitotic proliferation of local mature EC and vessel wall-resident stem cells [2], or by the recruitment of endothelial progenitor cells (EPC) from the bone marrow (BM) [3], [4]. Given the fact that proliferation of mature EC is fundamentally limited by telomere shortening and cellular senescence [5], [6], endothelial monolayer requires constant rejuvenation by EPC. In man, EPC have been defined as circulating CD34^+^ progenitor cells (PC) expressing the kinase insert domain receptor, a minor cell population that constitutes less than 0.004% of the circulating mononuclear cell fraction [7]. Numerous reports have shown an inverse relation between the number and/or function of circulating CD34^+^ cells and adverse metabolic and hemodynamic risk factors for cardiovascular disease [8]–[10].

The process of (E)PC mobilization is tightly controlled by activation of matrix metallopeptidase 9 (MMP9; EC 3.4.24.35) via the eNOS/NO/cGMP/MMP9 signaling pathway in BM [11]–[14]. Although the relevance of upstream endothelial nitric oxide synthase (eNOS; EC 1.14.13.39) activity for EPC mobilization and function is well-established [15]–[17], unequivocal detection and quantification of basal nitric oxide (NO) production in freshly-isolated BM cells has not yet been reported presumably due to the low concentration (nM range) and short lifespan (<1 ms) of NO radicals in vivo [18]. Furthermore, BM cell suspensions are heterogeneous mixtures of different cell types at various stages of differentiation, of which but a small fraction are capable to produce NO. In the present study, we developed a protocol for electron paramagnetic resonance (EPR) spin trapping of free NO radicals in rat BM cell suspensions. With this technique we confirmed that eNOS is the dominant source of NO in BM. Since hypertension is closely associated with endothelial dysfunction and impaired (E)PC mobilization [19], we compared NO level in the BM of normotensive and hypertensive rats. Further, with an adapted assay for human MMP9 activity [20], we quantified MMP9 bioavailability in these samples. Several publications [16], [17] have reported improved (E)PC mobilization and function after prolonged pharmacological enhancement of eNOS expression with AVE9488. The authors proposed that impaired function and mobilization of (E)PC correlates with NO levels in the BM. Here we confirm such close correlation between NO production and MMP9 levels in the BM of hypertensive as well as normotensive rats.

## Materials and Methods

### Deoxycorticosterone acetate-salt model of hypertension

The experimental protocol was conducted in accordance with institutional guidelines and approved by the Ethics Committee on Experimental Animal Welfare of Maastricht University (The Netherlands). To examine the effect of hypertension on NO production in BM we used 8 male and 4 female outbred Wistar rats purchased from Charles River (The Netherlands). Upon arrival, 9 weeks old animals with a body weight ranging from 226 to 250 gram were housed in groups of 2 animals per cage. Before experimentation, rats were allowed to acclimatize to the animal room for 7 days under controlled temperature and humidity conditions with an alternate 12:12 hr light-dark cycle. During the experiments rats had free access to standard rat chow ssniff R/M-H (ssniff Spezialdiäten GmbH, Germany).

All rats underwent left unilateral nephrectomy and, after 1 week of recovery, a silicon pellet (Sylgard 184 silicone elastomer base; Dow Corning, Midland, MI, US) containing 100 mg of 11-Deoxycorticosterone acetate (DOCA; Sigma, Germany) was implanted subcutaneously. Both procedures were performed under 1.5–4.0% isoflurane (IsoFlo; Abbott) anesthesia. The rats had *ad libitum* access to high-salt drinking water containing 0.9% NaCl (Sigma, Germany) and 0.2% KCl (Sigma, Germany) throughout the course of the experiment. Corresponding control rats were implanted with a subcutaneous silicon pellet without DOCA and these rats received regular tap water to drink. To record mean arterial pressure (MAP), after 6 weeks, heparinized (5 U/ml; Leo pharmaceuticals, Denmark) indwelling polyethylene catheters were introduced in a femoral artery and advanced into the lower abdominal aorta under 3–4% isoflurane anesthesia. After 2 days of recovery, the arterial catheter was connected to a pressure transducer (Micro Switch 150 PC) and its output was sampled at 2.5 kHz. MAP was derived from these signals using the IDEEQ data-acquisition system (instrument services, Maastricht University). Afterwards animals were sacrificed under 3–4% isoflurane anesthesia by exsanguination and the femurs, humeri and tibias were taken from each rat to isolate BM cell suspensions for analyses of NO and MMP9.

### BM cell isolation and treatment

BM cells were harvested at atmospheric oxygen level (ca 21%) by flushing isolated rat limb bones with warm (37°C) 20 mM HEPES (4-(2-hydroxyethyl)-1-piperazineethanesulfonic acid, Sigma, Germany) buffer (pH 7.4) supplemented with 5 mM glucose (LUMC pharmacy, the Netherlands) and 2.5 mM CaCl2 (Sigma, Germany) using a syringe. The six limb bones of a single rat typically delivered 400 (384±169) million white blood cells (WBC) as counted with a Beckmann Coulter Ac T system. The BM cell suspension from every rat was split into several different samples and used for MMP9 and NO detection after cell treatment with or without NOS activators and/or inhibitors.

If applicable, calcium ionophores A23187 (Sigma Aldrich, Germany, 10 µM final) or ionomycin (Sigma, Germany, 10 µM final) were used to activate constitutive NOS isoforms. As a nonselective NOS inhibitor, 500 µM *N*_ω_-Nitro-L-arginine methyl ester (L-NAME; Sigma, Germany) was used.

### Identification of NOS isoforms that contribute to NO production in the BM of Lewis rats

The experimental protocol was conducted in accordance with institutional guidelines and approved by the Ethics Committee on Experimental Animal Welfare of Leiden University Medical Center (The Netherlands). To identify the contribution of the different NOS isoforms to BM-derived NO production we used inbred Lewis rats (Harlan Laboratories, Boxmeer, The Netherlands). Animal characteristics, routine housing conditions and the harvesting of BM cells were performed as described above.

We applied the selective NOS inhibitor (N-(3-aminomethyl)benzyl)acetamidine (1400 W, Alexis Biochemicals, USA) at 2 concentrations: 0.1 µM - for inhibition of inducible NOS (iNOS) only, 10 µM - for inhibition of iNOS and neuronal NOS (nNOS) simultaneously. The effect of 1400 W on NO production in the BM was investigated in triplicates.

For proper NOS function, BM suspensions of Lewis rats were supplemented with 20 µM L-arginine (Sigma Aldrich, Germany), 10 µM (6R)-5,6,7,8-tetrahydrobiopterin ((6R)-BH_4_; Sigma Aldrich, Germany), 125 µM ß-nicotinamide adenine dinucleotide phosphate (NADPH; Alexis Biochemicals, USA), 10 µM flavin adenine dinucleotide (FAD; Sigma Aldrich, Germany) and 10 µg/ml of calmodulin (Sigma Aldrich, Germany).

In appropriate cases, constitutive NOS isoforms (eNOS and nNOS) were activated with 10 µM calcium ionophore A23187. Alternatively, 500 µM L-NAME was used to inhibit all NOS isoforms.

### Spin trapping of NO in BM suspensions

NO trapping in BM cell suspensions was done according to the protocol previously published [21]. It involves addition of Fe^2+^-citrate (10 µM final; FeSO_4_ and trisodium citrate, Sigma Aldrich, Germany) and sodium diethyldithiocarbamate (DETC, 2 mM final, Sigma Aldrich, Germany) to produce the lipophilic Fe-DETC complex that traps the free NO radicals.

After 30 min incubation at 37°C under an atmospheric oxygen level and 5% CO_2_, the suspension was placed on ice to terminate enzymatic NOS activity. The paramagnetic trapping adducts are mono-nitrosyl-iron complexes (MNIC, or NO-Fe^2+^-DETC) and located in the apolar cellular fraction that was harvested by 10 minutes of centrifugation at 4°C and 300×g. The pellet with the cellular fraction was resuspended in strong HEPES buffer (150 mM, pH 7.4), topped up to a final volume of *ca* 400±20 µl and snap frozen in liquid nitrogen as a small frozen column of 4.8 mm diameter for EPR assay.

The EPR spectra were measured on a modified X-band ESP300 spectrometer (Bruker BioSpin, Rheinstetten, Germany) operating with 20 mW microwave power. These frozen samples were carefully centered in a Bruker ER4103TM cylindrical cavity operating in TM_110_ mode with unloaded Q∼10.000. The sample temperature was kept at 77°K with a quartz liquid finger Dewar filled with liquid nitrogen. The magnetic field was modulated at a frequency of 100 kHz with 5 G amplitude. The detector gain was 2×10^5^, time constant 82 ms, and ADC conversion time 82 ms. Up to four field sweeps were accumulated to improve signal to noise. With these spectrometer settings, the detection limit was *ca* 10 pmol MNIC [21]. The MNIC yields in the tissue samples were quantified by comparing the EPR intensity of the triplet structure with that of frozen reference samples of paramagnetic NO-Fe^2+^-MGD complexes (10 µM) in PBS buffer. This comparison was done at identical temperature using the same hardware, microwave power and spectrometer settings (except for amplifier gain). This procedure affords an accuracy of the MNIC yields of *ca* 10%.

The sample reduction with dithionite (50 mM) was required [22] to suppress the signals from Cu^2+^–DETC complexes, that are usually visible in EPR spectra of biological samples. In unreduced state, these signals dominate and overlap with the EPR spectrum of the true MNIC, thereby obscuring its observation [23]. The reduced tissues show undistorted spectra of NO-Fe^2+^-DETC with their characteristic triplet, which is possible to quantify. Given our modest MNIC yields, reduction with dithionite was absolutely required for quantification of MNIC in BM.

### MMP9 activity assay

To measure MMP9 activity in the supernatant of BM cell suspension we developed a rat-specific assay that allows us to quantify total as well as active MMP9. The assay was adapted from a human-specific MMP9 immunocapture activity assay [20] by modifying the MMP9 standard and optimization of its activation by *p*-aminophenylmercuric acetate (APMA). It has a detection range from 4 to 16.000 pg MMP9 per ml.

In short, rat BM supernatants were incubated overnight with anti-MMP9 antibodies (S22-2, QuickZyme Biosciences, Leiden, The Netherlands), coated to a high binding 96-well plate (polystyrene stripwell plate; Corning B.V. Life Sciences, The Netherlands). To detect total MMP9 activity, bound MMP9 was activated with 0.5 mM APMA at 37°C for 4 hours. As a standard, serial dilutions ranging from 0 to 16 ng/ml of recombinant rat pro-MMP9 (R&D systems, Minneapolis, USA) were used and activated in parallel with rat BM samples. Active MMP9 was detected with a colorimetric enzymatic assay using the modified enzyme Ukcol and the chromogenic peptide substrate S-2444 [20]. The resultant color was read at 405 nm in a microplate spectrophotometer immediately and every 10–20 minutes during the next 4 hours, while the plate was incubated at 37°C. The absolute concentration of MMP9 in a sample was determined by interpolation from a calibration curve.

### Statistical analysis

All values are presented as mean with standard deviation. Treated and untreated groups were compared using the non-parametric Mann-Whitney U test, as appropriate for skewed data. Correlations were analyzed by Spearman's correlation method. All statistical calculations were performed using GraphPad Prism software (La Jolla, California, USA). P≤0.05 was considered to denote statistical significance.

## Results

### Enzymatic generation of NO is detectable in rat BM

NO radicals are implicated in many (patho)physiological processes, but hard to detect in living tissues due to low concentration and short lifespan. Therefore, most studies use downstream metabolites like NO_2_^−^ (Griess assay), NO_2_ (chemiluminescent assay) or N_2_O_3_ (diaminofluorescein assay) as experimental proxies for ‘true’ NO [24]. In biological systems the latter may be detected with EPR spectroscopy [25] and spin trapping with Fe-DETC complexes, as this affords selectivity for detection of free NO radicals.

**Figure 1:**
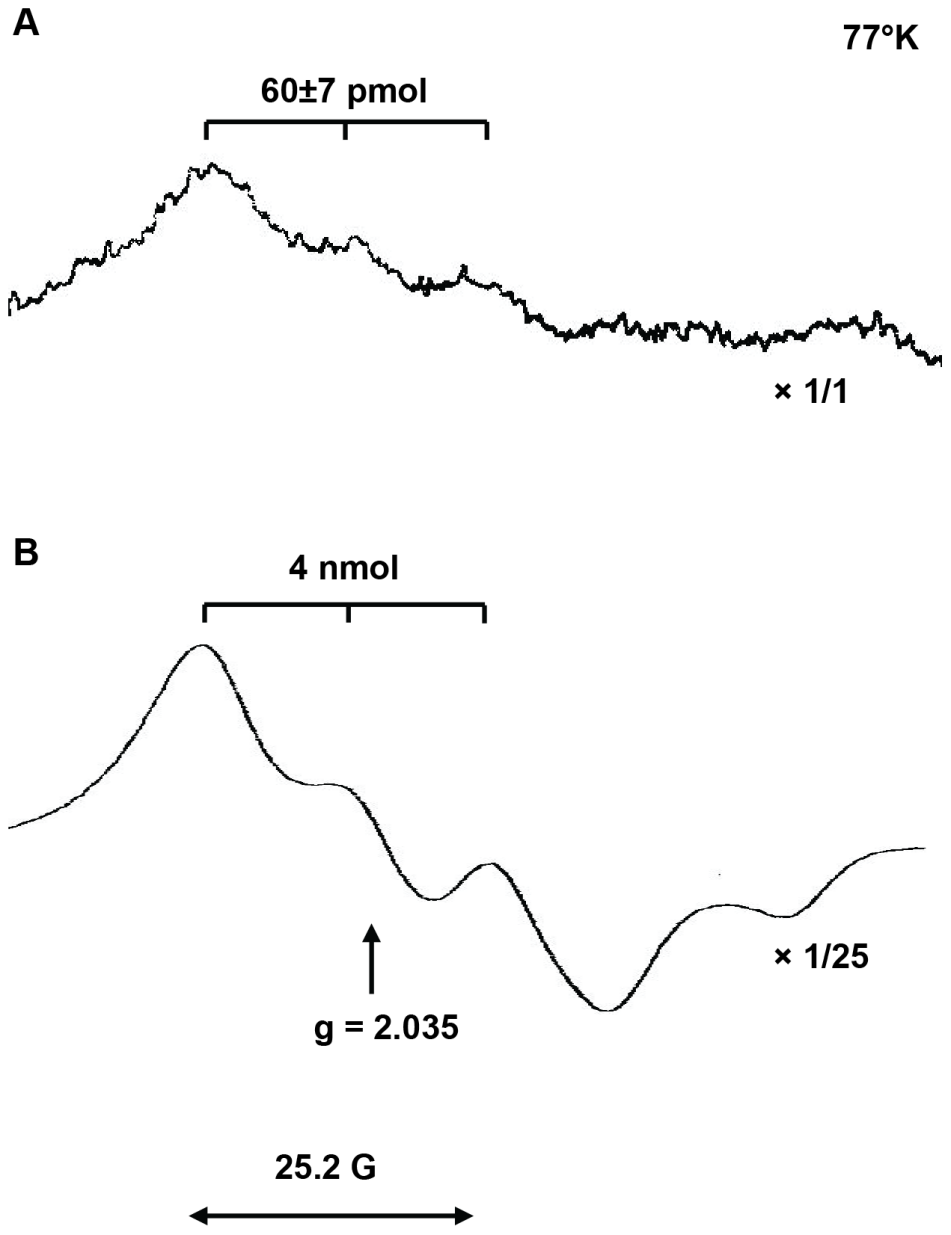
X-band EPR spectra of MNIC at 77°K. (A). The EPR spectrum from BM cell suspension of *ca* 100 mln cells. These cells were collected from a DOCA-treated Wistar male rat and stimulated for NO production with Ca-ionophore A23187. The intensity of the spectrum was not changed and was quantified as 60±7 pmol MNIC. (B) The reference spectrum of a strong calibration sample with 4 nmol MNIC, showing the characteristic triplet structure centered around g = 2.035 with a hyperfine splitting of 25.2 G = 2A_N_^14^. The intensity of the reference spectrum was scaled down by a factor 25.

We used this method to quantify NO levels at 37°C in buffered neutral solution (pH = 7.4) open to ambient air. Such conditions preclude potential artefactual NO release from S-nitrosothiols, endogenous nitrite anions and organic nitrates [21], [26]. After 30 minutes of trapping in freshly isolated BM cell suspensions, samples were snap frozen and the trapping yield of MNIC was quantified with EPR spectroscopy at 77°K. Figure 1A shows the X-band EPR spectrum of a BM sample (100×10^6^ WBC) collected from a DOCA-treated Wistar male rat and stimulated for NO production with Ca-ionophore A23187. This EPR spectrum is quantified as 60 pmol MNIC and unambiguously identified from its characteristic triplet lineshape, g-factor (g = 2.035) and triplet-hyperfine coupling constant A_N_^14^ = 12.6G. Figure 1B shows the spectrum of a strong calibration sample containing *circa* 4 nmol MNIC.

**Figure 2:**
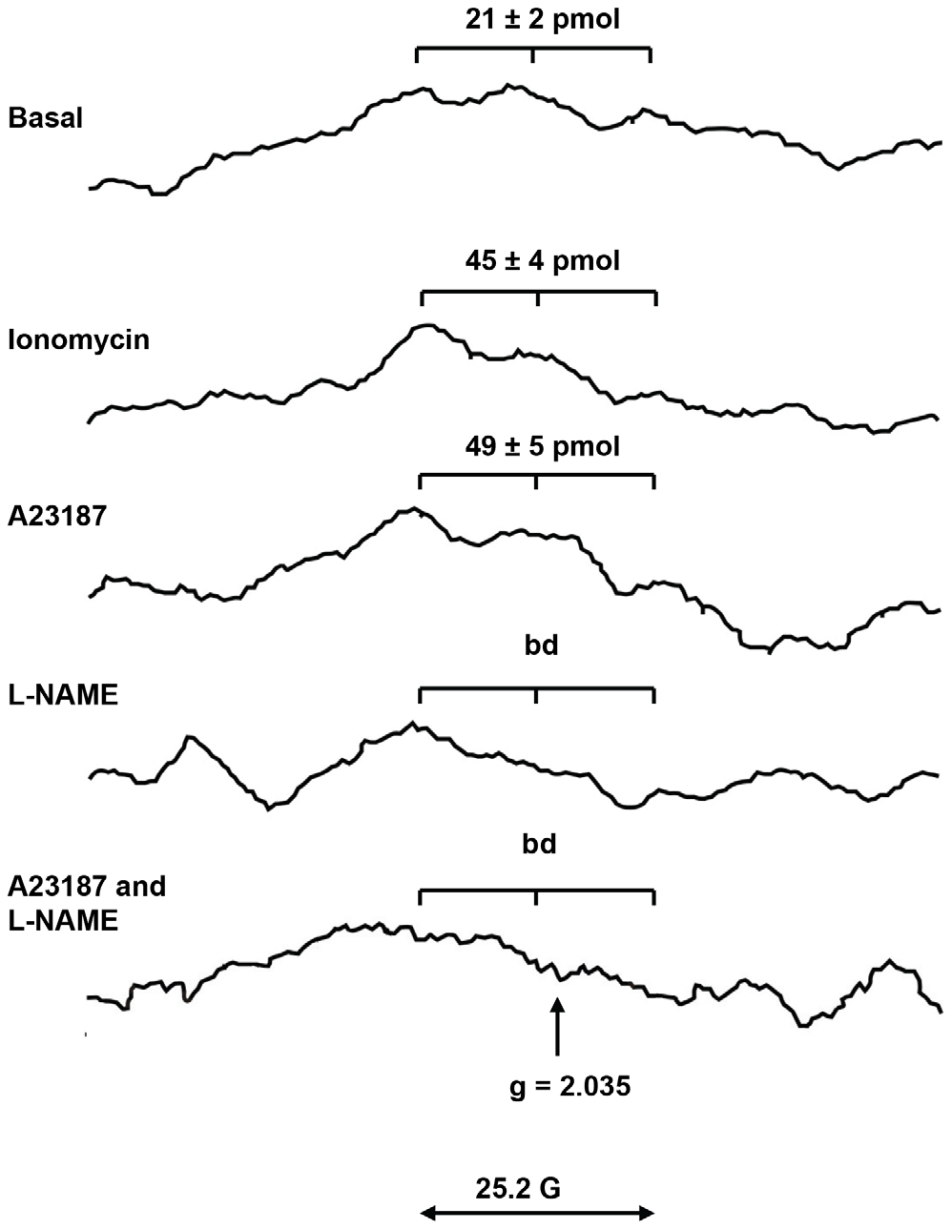
The effect of NOS inhibitors and/or activators on NO level in BM of Wistar rats. X-band EPR spectra at 77°K of frozen BM cell suspensions from Wistar rats after 30 min of NO trapping with Fe^2+^-DETC. The samples (53±2×10^6^ BM cells) were reduced by 50 mM dithionite to remove the overlapping signal from paramagnetic Cu^2+^-DETC. A basal yield of 21±2 pmol MNIC is obtained in absence of stimulus (top), clearly recognizable by its characteristic hyperfine triplet structure at g = 2.035. Stimulation with ionomycin or A23187 calcium ionophores raises the MNIC yields to 45±4 and 49±5 pmol. In presence of the NOS inhibitor L-NAME, the MNIC yields remained below the detection threshold of *ca* 10 pmol. The EPR spectra show significant background signals not related to MNIC, as visible in the bottom spectra. Such background signals derive from paramagnetic iron centers, and some residual Cu^2+^-DETC as commonly found in biological samples [18].

To confirm that detected NO is a result of activity of NOS enzymes, we quantified MNIC in BM samples of Wistar rats (53±2×10^6^ WBC per sample) in the presence and/or absence of NOS activators and inhibitors (Figure 2). In absence of any stimulus, a basal yield of 21±2 pmol MNIC is visible above the instrumental noise with a S/N ratio of 2–3. Upon stimulation with 10 µM ionomycin or 10 µM A23187, that increase intracellular calcium levels and activate the constitutive eNOS and nNOS isoforms [27], MNIC yields were raised to 45±4 and 49±5 pmol respectively. Moreover, in presence of 500 µM L-NAME, a non-selective NOS inhibitor, MNIC yields remained below the detection threshold of 10 pmol both in the absence and presence of Ca-ionophore. These experiments demonstrate conclusively that the formation of MNIC adducts in rat BM arises from enzymatic arginine–citrulline conversion by NOS. The specificity of Fe-DETC complexes for free NO radicals affords the first direct confirmation of the production of NO radicals *per se* in BM.

### Endothelial NOS is the dominant source of NO in BM

Next, we aimed to define the identity of the NOS isoforms that contribute to the basal NO production in rat BM. Along with eNOS, at least two other NOS isoforms could potentially contribute NO in the BM–iNOS and nNOS. Unfortunately there are no 100% isoform-specific NOS inhibitors. Among others, 1400 W stands out as highly isoform-selective NOS inhibitor that can be used *in vitro* and *in vivo*, with K_i_ values of 0.007 µM, 2 µM, and 50 µM for purified human iNOS, nNOS, and eNOS respectively [28]. In rat aortic rings, 1400 W has been found to be at least 1,000-fold more potent against iNOS compared to eNOS [28]. Therefore, we used 1400 W for an identification of active NOS isoforms in rat BM.

Since the MNIC yields in BM cell suspension of 53±2×10^6^ WBC had S/N ratios of 2–3, we decided to increase the sample size to the equivalent of 100×10^6^ WBC per sample and as a result, the MNIC spectra became more apparent. Also, given the smaller standard deviation for an inbred over outbred rat strain, we used Lewis rats to investigate the NOS isotype-specific contribution to NO production in BM.

**Figure 3:**
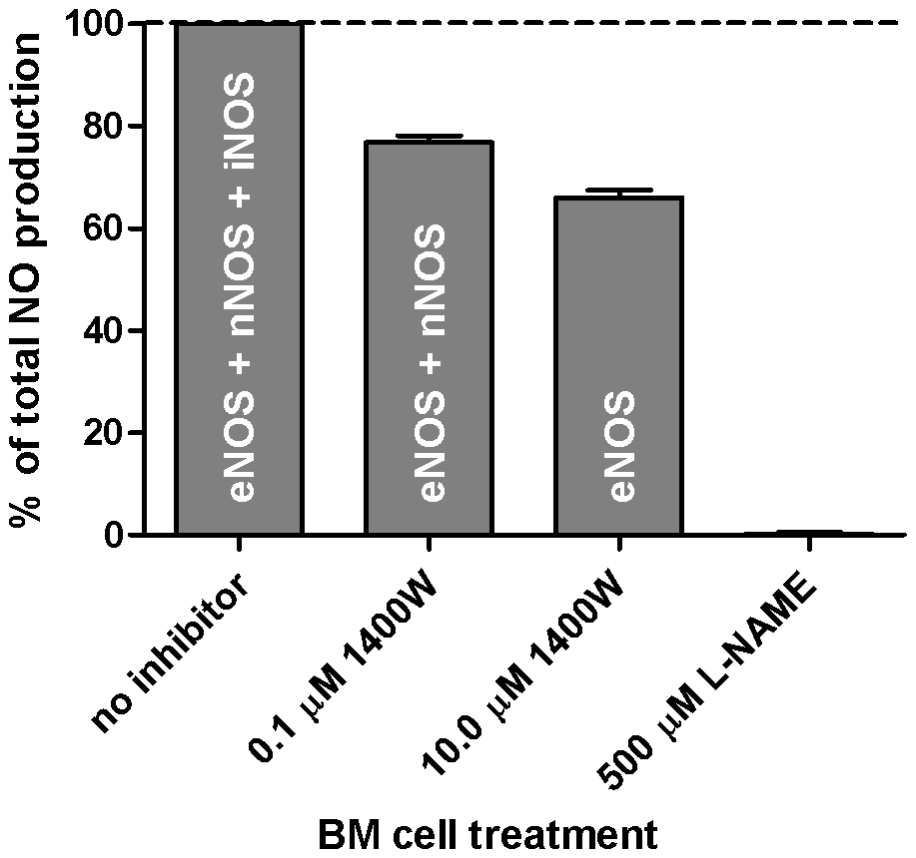
Relative contribution of NOS isoforms to basal NO production in BM of Lewis rats. The basal NO level that was obtained in absence of a stimulus was taken as 100%. Contribution of the NOS isotypes was determined using two concentrations of the NOS isoform-specific inhibitor 1400 W. 1400 W was used at final concentrations of 0.1 µM for iNOS inhibition and 10 µM for inhibition of both iNOS and nNOS. With L-NAME (500 µM, final column), the signal intensity was below the detection threshold. Values represent biological triplicates.

We examined MNIC yields in BM suspensions of Lewis rats at [1400 W] = 0.0 (basal), 0.1 and 10.0 µM. The results of three independent experiments show that without stimulation or inhibition, the basal NO production was 38±6 pmol MNIC per 10^8^ cells. We note that this Lewis yield is in good agreement with 21±2 pmol MNIC per 53±2×10^6^ cells previously found in Wistar rats. This basal yield was taken as 100% (Figure 3). Pretreatment of BM cells with 0.1 µM 1400 W reduced MNIC yield to 77±1% of basal. As only iNOS is inhibited by this low concentration of 1400 W, we estimate that iNOS contributes to approximately 23% of baseline NO levels. At [1400 W] = 10 µM, we find a further loss of 11±2% in MNIC yield, attributable to nNOS. Finally, the addition of 500 µM L-NAME completely abolished MNIC. Based on these yields, we conclude that the majority (*circa* 66%) of basal NO derives from eNOS, with smaller but non-negligible contributions from iNOS and nNOS.

### NO production in BM is affected by peripheral hypertension

Hypertension is strongly associated with chronic inflammation, oxidative stress and endothelial dysfunction in both humans and rats [29], [30]. Moreover, in both these species, circulating levels of EPC are reduced suggesting impaired BM (mobilization) function [19]. Therefore we tested whether peripheral hypertension might affect the eNOS and iNOS activity in BM and modify the NO bioavailability there. To this end, we induced hypertension in Wistar rats by unilateral nephrectomy followed by chronic leaching of DOCA-salt from an implanted silicon pellet [31], [32]. After 6 weeks of DOCA-treatment, MAP was increased to levels seen in severe peripheral endothelial dysfunction [33], [34] (table 1). In all control rats, the MNIC yields in the BM were above 20 pmol and clearly detectable with an average value of 38±16 pmol MNIC per 10^8^ cells. The MNIC yields in the BM of DOCA-hypertensive rats were lower and harder to quantify. In two out of six cases MNIC yields remained below the detection limit of 10 pmol. In other BM samples of hypertensive rats MNIC yield exceeded 20 pmol with a mean value circa 29±7 pmol per 10^8^ cells. With MNIC yield near the EPR detection limit and considerable variation between individual Wistar rats (an outbred strain), the averaged NO yields present with large error margins. These large margins make NO levels in BM of DOCA group not significantly different from those in the control group.

**Table 1:** MAP and BM-derived MNIC yields in control and hypertensive Wistar rats.

Parameters	Control	DOCA	P	units
	rats	rats		
	(n = 6)	(n = 6)		
MAP	114±7	173±7	<0.01	mm Hg
MNIC: basal NOS activity	38±16	29±7*	0.45	pmol/10^8^ cells
MNIC: A23187-stimulated NOS activity	61±27	60±24	0.84	pmol/10^8^ cells
MNIC: L-NAME-inhibited NOS activity	b.d.	b.d.		pmol/10^8^ cells

b.d.: below detection

* Two BM samples, where MNIC remained below the detection threshold are excluded from this average.

As shown in table 2, basal MNIC yields from inbred Lewis and outbred Wistar rats are in full agreement, with smaller standard deviation for the genetically homogenous inbred strain. This result confirms that EPR dosimetry can be used for the quantification of NO provided MNIC yields exceed *ca* 20 pmol.

**Table 2:** MNIC yields (pmol/10^8^ cells) in rat BM suspensions and EC cultures.

MNIC yields	Wistar BM	Lewis BM	HUVEC*	bEnd.3*
basal NOS activity	38±16	38±6	395±40	1470±150
A23187-stimulated NOS activity	61±27	65±7	710±150	5330±600

* Technical footnote: Since proper eNOS activity requires EC attachment and cell-cell contact, NO trapping was done on monolayers of HUVEC and bEnd.3 cells firmly attached to the bottom of a culture T_75_ flasks, as described previously [21]. A single flask with *ca* 7.5×10^6^ confluent bEnd.3 cells has adequate signal to noise ratio [21], whereas HUVEC, having less eNOS, requires at least two flasks with total 15×10^6^ cells.

Interestingly, pre-incubation with Ca-ionophore A23187 raises BM-derived NO levels about twofold in both strains (Wistar DOCA 60±24 pmol MNIC per 10^8^ cells; Wistar controls 61±27 pmol MNIC per 10^8^ cells; Lewis 65±7 pmol MNIC per 10^8^ cells; tables 1 and 2). Taken together, these data suggest that hypertensive NO deficiency may reflect differences in posttranslational regulation of NOS rather than differences in NOS expression *per se*.

### NO levels in rat BM are physiologically relevant

**Figure 4:**
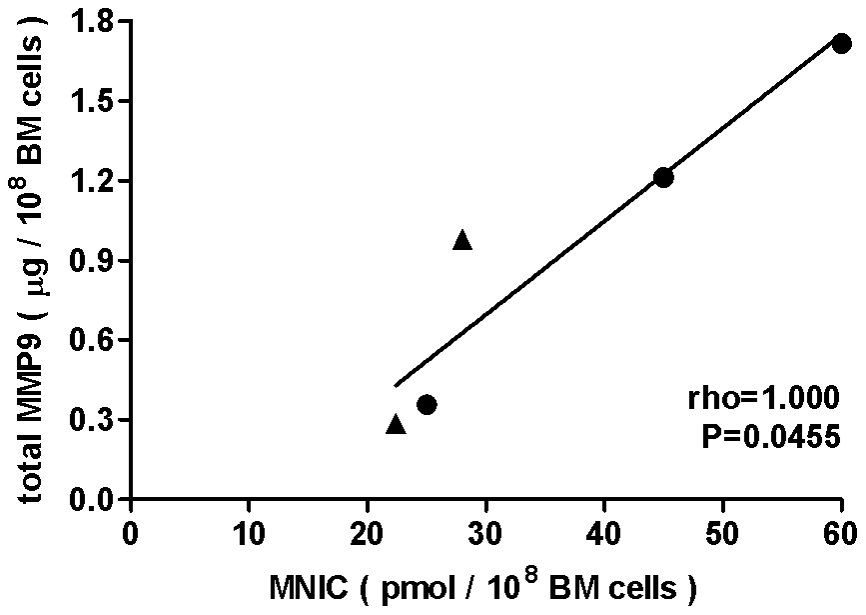
Spearman rank correlation between NO and MMP9 levels in BM of Wistar rats. A positive and statistically significant (0.01<P<0.05) Spearman correlation was obtained between the basal NO levels and total MMP9 amount in BM of DOCA-salt induced hypertensive (▴) and control (•) Wistar rats. MNIC yields were detected in BM samples of 100×10^6^ cells, total MMP9 amount was quantified in the supernatants from the same BM samples. Errors in MNIC yields and MMP9 (not indicated) are ca 10% and 5% respectively.

Given the importance of the eNOS/NO/MMP9 signalling cascade for (E)PC mobilization from BM [11], [13], [35], we raised the question whether NO deficiency in the BM of hypertensive rats reflects in activity of downstream regulatory proteins like MMP9, the presumptive rate limiting factor for (E)PC mobilization. Adapting a human MMP9 activity assay to rodent biological fluids/materials, we quantified MMP9 in BM of hypertensive and control rats as 0.9±0.4 µg per 10^8^ cells and 1.1±0.6 µg per 10^8^ cells, respectively. Given the large errors, these averages appear not statistically different (p = 0.41). However, the very tight correlation between MMP9 and MNIC in individual rats (Figure 4, Spearman rho = 1.000 and p<0.05) corroborates our hypothesis that inadequate (E)PC mobilization in hypertension results from compromised NOS activity.

## Discussion

Here, we give the first confirmation of the enzymatic release of free NO radicals in rat BM. Our data show that in a healthy state eNOS is a dominant source of NO in BM. Additionally, in BM of experimental hypertensive rats the NO level is lower than in healthy controls and directly relate to the production of MMP9, supporting observations that eNOS deficiency impairs the (E)PC mobilization.

EPR is widely used in biosystems for detection and quantification of paramagnetic complexes with trapped NO radicals. At the same time, the EPR spectra of frozen mammalian tissues are usually dominated by Cu^2+^–DETC complexes, which overlap with MNIC, obscuring the latter [23]. Reduction with dithionite allowed us to suppress the Cu^2+^–DETC signal and make MNIC spectra visible [22]. In addition to removing the overlapping Cu-DETC signal, reduction with dithionite is known to enhance MNIC yields in vivo and in vitro, thereby enhancing the sensitivity of the NO trapping considerably [36], [37].

A paramagnetic MNIC adducts in living tissues is usually taken as evidence for free NO radicals. This is not strictly true, since endogenous S-nitrosothiols, nitrite and organic nitrates could be an alternative non-enzymatic sources of NO [18].

There are several mechanisms of free NO release from endogenous S-nitrosothiols that can occur *in vitro*. For example, decomposition of S-nitrosothiols into NO and a thiyl radical occurs spontaneously by thermolysis or photolysis, or by the catalytic activity of reduced transition metal ions like Cu^+^ or Fe^2+^ [18]. Alternatively, high concentrations of low molecular weight S-nitrosothiols may even nitrosylate iron complexes like the Fe-DETC traps used here, by direct transfer of the NO-moiety [38]. However, at physiological S-nitroso levels, the reaction rate of such transnitrosation or decomposition is six orders of magnitude smaller than the trapping rate of free NO radicals and it makes this alternative pathway of MNIC formation negligible in living tissues [18].

Again, endogenous nitrite anions are prone to be reduced to free NO under hypoxic conditions by a plethora of ubiquitous mammalian enzymes like xanthine oxidase or cytochrome P450 [26]. Therefore, we should carefully consider such alternative pathways for release of NO and formation of MNIC.

It is known that DETC ligands may cause confounding artefacts by inhibiting NFkB and Cu-Zn-SOD. As discussed at length [36], such artefacts are not significant under the conditions used in this work.

In our case it is justified to attribute the observed MNIC yields to free NO radicals deriving from enzymatic NOS activity in the BM suspension. First, the NO trapping experiments were carried out under normoxic conditions, so that the hypoxic pathways described above cannot operate. Second, our experiments were performed in buffered neutral solutions (pH = 7.4), where spontaneous acidic decomposition of nitrite cannot occur. In addition, the complete abolition of MNIC by NOS inhibitors confirms that such artefactual pathways do not generate significant quantities of MNIC in our experiments. Therefore, we attribute the formation of detected MNIC adducts unequivocally to arginine–citrulline conversion by NOS.

Such sensitivity and specificity of MNIC for NO makes the EPR trapping technique uniquely suited to assess endogenous NO levels *in vivo* or in freshly isolated tissue samples as BM.

As a cautionary note, we apprise that the MNIC should not be equaled with NO production, since only a minor fraction of NO is trapped by Fe-DETC and the majority of NO is lost via other reaction pathways. Therefore, the MNIC yield is only a firm lower bound for the true NO yield. In practical biological settings of laboratory animals or cell cultures, only about 10% to 20% of NO gets trapped into MNIC [18].

Next to a first direct confirmation and quantification of NO production in freshly isolated BM, we found that the basal, i.e. unstimulated, NO levels in inbred Lewis rats (n = 3, 38±6 pmol MNIC per 10^8^ cells) are identical to those in outbred Wistar rats (n = 6, 38±16 pmol MNIC per 10^8^ cells), with a smaller standard deviation for the genetically identical Lewis rat strain. In the presence of a Ca-ionophore, NO production almost doubled in both rat strains (Table 2). These findings appear plausible and attest to the reliability of NO trapping.

To better appreciate the magnitude of MNIC production in BM suspensions, we compared it with MNIC yields in two types of cultured EC. As shown in Table 2, non-stimulated murine microvascular brain EC (bEnd.3 cells) release *circa* 1470±150 pmol MNIC per 10^8^ cells [21]. We find that freshly isolated and cultured primary human umbilical vein EC (HUVEC) release fourfold less NO. This is consistent with previous observations that the bEnd.3 cell line expresses relatively high levels of eNOS and displays a copious basal release of NO [21]. Nevertheless, the fact that the BM suspensions, although heterogeneous mixtures of WBC, hematopoietic cells at various stages of differentiation, adipocytes, fibroblasts and EC, still achieve nearly 10% of HUVEC yields, indicates that basal BM-derived NO levels are quite significant.

Based on the calcium dependence and the selective sensitivity for the isotype-specific NOS inhibitor 1400 W, we conclude that eNOS is the major contributor (66%) to the NO production of BM cell suspensions.

Inducible NOS produces NO at a high rate in a calcium-independent manner and is conventionally thought to be expressed in response to inflammatory mediators [39]. However, we found that iNOS is responsible for nearly one quarter (23%) of basal NO production in rat BM. This serendipitous finding might surprise at first, however a substantial basal iNOS activity in BM is not implausible since previous studies identified iNOS RNA in bone and in BM-derived PC even in the absence of inflammation [40], [41]. It was reported that normal osteoblast maintenance requires some degree of iNOS activity [42] and therefore expression of iNOS in BM cell suspension is expected. Our experiments confirm that iNOS contributes significantly to basal NO levels in BM, even in the absence of inflammation.

Given the dominant contribution of eNOS to basal NO production in rodent BM and the reduced amount and/or function of (E)PC in hypertension or other cardio-vascular disease [8], [10], [16], [17] we expected to detect reduced eNOS derived NO production in the BM of DOCA-hypertensive rats. On the other hand, hypertension is known to be associated with a chronic inflammatory state [43] inducing an oxidative stress that might affect the intracellular redox environment [8] and production of iNOS-derived NO. Our data confirmed that the BM of DOCA-hypertensive rats produces lower MNIC yields than the BM of control rats. This supports the hypothesis that, in patients at risk for cardiovascular disease such as hypertension, peripheral endothelial dysfunction extends to the bone marrow and may result in reduced NO bioavailability and, consequently, impaired MMP9-dependent (E)PC mobilization [11], [19]. Indeed, in the individual BM samples we found a tight correlation between NO and MMP9 (Figure 4), the upstream and downstream mediators of BM cell mobilization, respectively.

Finally, the fact that Ca-ionophores elevate NO levels even in the BM of hypertensive rats provides proof of principle that eNOS activity may be recovered by pharmacological intervention.

We strongly believe that detection of NO by EPR may serve as a highly valuable tool to assess the effect of therapeutic strategies aimed at correcting NOS function and (E)PC mobilization from BM in experimental animal models of cardiovascular disease. Furthermore, our approach for NO detection may be more broadly applicable as the NOS/NO/sGC/cGMP/PKG/MMP9 pathway is active in other organs and tissues and involved in (patho)physiological processes such as ischemia/reperfusion injury, organ fibrosis or synaptic plasticity in the nervous system.

### Innovation

With NO spin trapping, we demonstrate significant release of NO radicals in BM suspensions of rats. The NO production reflects enzymatic activity of NO synthases, with significant contributions from iNOS even under non-inflammatory conditions. We observe a close correlation between NO and MMP9 in BM, proving the principle that MMP9 levels may be modified by upstream pharmacological intervention in the NOS/NO/MMP9 pathway. Given the role of MMP9 in mobilization of hematopoietic stem and (endothelial) progenitor cells, our findings confirm NO levels in the BM as a potential modifiable upstream target to promote E(PC) mobilization in cardiovascular disease.
